# The Development of Diet-Induced Obesity and Glucose Intolerance in C57Bl/6 Mice on a High-Fat Diet Consists of Distinct Phases

**DOI:** 10.1371/journal.pone.0106159

**Published:** 2014-08-29

**Authors:** Lynda M. Williams, Fiona M. Campbell, Janice E. Drew, Christiane Koch, Nigel Hoggard, William D. Rees, Torkamol Kamolrat, Ha Thi Ngo, Inger-Lise Steffensen, Stuart R. Gray, Alexander Tups

**Affiliations:** 1 Rowett Institute of Nutrition and Health, University of Aberdeen, Aberdeen, United Kingdom; 2 Department of Animal Physiology, Faculty of Biology, Philipps University Marburg, Marburg, Germany; 3 Musculoskeletal Research Programme, Institute of Medical Sciences, University of Aberdeen, Aberdeen, United Kingdom; 4 Department of Food, Water and Cosmetics, Division of Environmental Medicine, Norwegian Institute of Public Health, Oslo, Norway; University of East Anglia, United Kingdom

## Abstract

High–fat (HF) diet-induced obesity and insulin insensitivity are associated with inflammation, particularly in white adipose tissue (WAT). However, insulin insensitivity is apparent within days of HF feeding when gains in adiposity and changes in markers of inflammation are relatively minor. To investigate further the effects of HF diet, C57Bl/6J mice were fed either a low (LF) or HF diet for 3 days to 16 weeks, or fed the HF-diet matched to the caloric intake of the LF diet (PF) for 3 days or 1 week, with the time course of glucose tolerance and inflammatory gene expression measured in liver, muscle and WAT. HF fed mice gained adiposity and liver lipid steadily over 16 weeks, but developed glucose intolerance, assessed by intraperitoneal glucose tolerance tests (IPGTT), in two phases. The first phase, after 3 days, resulted in a 50% increase in area under the curve (AUC) for HF and PF mice, which improved to 30% after 1 week and remained stable until 12 weeks. Between 12 and 16 weeks the difference in AUC increased to 60%, when gene markers of inflammation appeared in WAT and muscle but not in liver. Plasma proteomics were used to reveal an acute phase response at day 3. Data from PF mice reveals that glucose intolerance and the acute phase response are the result of the HF composition of the diet and increased caloric intake respectively. Thus, the initial increase in glucose intolerance due to a HF diet occurs concurrently with an acute phase response but these effects are caused by different properties of the diet. The second increase in glucose intolerance occurs between 12 - 16 weeks of HF diet and is correlated with WAT and muscle inflammation. Between these times glucose tolerance remains stable and markers of inflammation are undetectable.

## Introduction

Obesity and related metabolic disorders are largely the result of overconsumption of energy dense foods, high in sugar and long chain saturated fats. Diet-induced obesity leads to insulin insensitivity and numerous studies have shown that obesity and insulin insensitivity are related to the presence of low-grade inflammation [1], [2]. Further evidence for the role inflammation plays in obesity comes from studies where inflammation is either inhibited, leading to the prevention of insulin insensitivity and reductions in weight gain [3]–[5], or the inhibition of anti-inflammatory pathways which increases weight gain and the development of the metabolic syndrome [6]. While it is well established that dietary long chain saturated fatty acids cause insulin insensitivity and obesity [7], there is some debate as to the role of lipid induced increases in gut permeability and subsequent leakage of gut bacterial lipopolysaccharide (LPS) [8], as opposed to the role of lipid overload and ectopic fat deposition [9], [10] in obesity related inflammation.

Most studies have focused on the development of obesity and insulin insensitivity in rodent models of either genetic or diet-induced obesity after several weeks on a HF diet. These studies have shown that insulin insensitivity is related to cellular inflammation involving the JNK1 and IKKβ-NFκB cascade [11], [12], while relatively few studies have focused on the rapid induction of insulin insensitivity seen within 1 week of HF diet. Nonetheless, studies of early responses to a HF diet are emerging with one study showing that insulin insensitivity after 1 week of HF diet in the C57Bl/6 mouse is the consequence of insulin insensitivity in the vascular endothelium [13], and another demonstrating that hypothalamic markers of inflammation are activated between 1 and 3 days on a HF diet [14]. One study looking at both short- and long-term HF diet induced insulin resistance found inflammation increased from day 1 of HF feeding onwards [15], but concluded after looking at the response to HF diet in three different immuno-compromised mouse models, that inflammation was not necessary for the development of short-term but necessary for long-term insulin resistance [15]. More recently, inflammation in WAT was shown to contribute to the early induction of insulin insensitivity [16], while in contrast a separate study argued for the role of lipid metabolite accumulation in tissues early in HF feeding [17]. Other studies using transcriptomic approaches have identified early inflammation in the liver together with gradually increasing inflammation in WAT after HF feeding in mice [18], [19]. Indeed, Kupffer cell activation has been demonstrated after 3 days on a HF diet and Kupffer cell ablation at this time can prevent the associated hepatic insulin insensitivity and reduce adiposity [20]. However, if ablation of Kupffer cells takes place after macrophage infiltration of WAT is established it does not improve metabolic health [21], arguing for a role of liver inflammation in the early, but not later, development of insulin insensitivity and adiposity.

In the present study we hypothesised that the initial stages of glucose intolerance are induced by the HF composition of the diet. Thus, our aim was to further investigate the timeline of the induction of glucose intolerance and inflammation, particularly the early, within days, response to a HF diet. We used plasma proteomics to detect what, if any, changes in major plasma proteins occurred and identified a transient acute phase response at this time. Furthermore, mice fed the HF diet matched to the caloric intake of the LF fed mice (PF) demonstrated that glucose intolerance seen after 3 days is attributable to diet composition in comparison to the high levels of acute phase proteins seen at 3 days which are attributable to the additional calories in the diet. In summary, our data show a rapid, transient induction of relatively high levels of glucose intolerance coincident with an acute phase response but these two physiological events appear to be caused by different mechanisms in C57Bl/6 mouse fed a HF diet. The peak in glucose intolerance seen within 3 days of HF diet is followed by a relative improvement in glucose tolerance which remains stable between 1 and 12 weeks on HF diet with undetectable levels of inflammation in most organs until between 12 and 16 weeks when gene markers of inflammation become apparent in WAT and muscle, but not in liver, and the animals become more glucose intolerant. These data argue that glucose intolerance and inflammation do not increase gradually on a HF diet but occur in distinct phases.

## Methods

### Ethics statement

All studies involving animals were licensed under the Animal (Scientific Procedures) Act of 1986 and had received approval from the Rowett Institute of Nutrition and Health's Ethical Review Committee.

### Animals

Male C57BL/6 mice, 12 weeks of age and lacking mutations in the NNT gene, which may result in impaired insulin secretion [22], (Harlan, Bicester, UK), were singly housed on grid floors and either fed a HF (60% of energy from fat and 5.2 kcal/g) or a LF diet (10% of energy from fat and 3.8kcal/g) (D12492 and D12450B, respectively; Research Diets, NJ, US) *ad libitum* for either 3 days or 1, 4, 8, 12 or 16 weeks. Please see http://www.researchdiets.com/opensource-diets/stock-diets/dio-series-diets for diet composition details. Also additional groups of mice were pair-fed the HF diet restricted to the caloric intake of the LF fed animals for 3 days or 1 week (PF). LF fed mice eat on average 3.18 g/day/mouse and HF fed mice eat 2.9 g/day/mouse. Thus, PF mice were given 2.3 g/day/mouse of the HF diet to match the caloric intake to that of LF fed mice. Single housing and grid floors were utilised to enable accurate measurement of food intake, to prevent coprophagia and stress due to dominance. However, it is recognised that single housing of mice may in itself cause stress and this is alleviated to a certain extent by the provision of environmental enrichment and housing mice close to each other. The design of the study recognises that at different ages mice will show different levels of gene expression and may react differently to diet composition. Food intakes and body weights were measured twice weekly and MRI scans (EchoMRI, Houston, TX, USA), providing body composition of conscious mice, were carried out at the beginning of the study, at 3 days, and weekly thereafter (n = 8–12).

### Glucose tolerance

Intraperitoneal glucose tolerance tests (IPGTTs) were carried out (n = 6–8) as a non-recovery procedure after fasting for 5 hours. A blood sample (0 mins) was taken prior to the intraperitoneal IP glucose injection (1.5 mg/g body weight). Subsequent blood samples were taken from the tail vein at 15, 30, 60 and 120 mins and measured using an Accu chek Aviva blood glucose monitor (Roche Diagnostics, Burgess Hill, UK). Area under the curve (AUC) was calculated by using the trapezoid rule [23].

### Immunohistochemistry

A separate group of animals were killed by cardiac puncture under terminal anaesthesia induced by Euthatal (sodium phenobarbital) at a concentration of 500 mg/Kg injected IP. Liver, epididymal WAT and the gastrocnemius muscles were fixed in 4% paraformaldehyde for immunohistochemistry and/or oil red O staining (n = 6–8). Tissue was frozen and cryosections cut for oil red O staining. For immunohistochemistry tissues were wax embedded, sections were cut and dewaxed before staining with a rat anti mouse F4/80 antibody (AbD Serotec, Kidlington, UK). Further steps were carried out using the Vectastain Elite ABC Kit according to the manufacturer's instructions (Vector Laboratories, Peterborough, UK). Analysis of microscope images was carried out using an Image Pro-plus system (Media Cybernetics, Silver Springs, MD, USA).

### Real-time PCR assays

Tissues were frozen in liquid nitrogen for real-time PCR. Total RNA was extracted from liver, WAT and ileum using an RNeasy Mini Kit (Qiagen, Crawley, UK) incorporating DNase digestion. Total RNA was isolated from gastrocnemius muscle using TRIzol reagent (Invitrogen/Life Technologies, Carlsbad, CA, US). Extracted total RNA was quantified using a NanoDrop Spectrophotometer (NanoDrop Technologies). Quality was assessed using an Agilent Bioanalyser (Agilent Technologies).

Complementary cDNA templates for real-time PCR assays were prepared from Superscript II (Invitrogen) reverse transcribed total RNA (0.5–2.5 µg). Taqman real-time PCR assays were performed using VIC and FAM reporter dyes. Taqman primer assays for *B2M* (Mm00437762_m1), 18S (part no. 4319413E), β-actin (*ACTB*) (Mm00607939_s1), *GAPDH* (Mm99999915_g1), *haptoglobin* (Mm00516884_m1), *CXCL1* (Mm00433859_m1), *F4/80* (Mm00802529_m1), *MRC-1* (Mm00485148_m1), *ARG* (Mm00475988_m1), *ITgax* (Mm00498698_m1), *IL-10* (Mm004439614_m1), *APOA4* (Mm00431814_m1), *IL-6* (Mm99999064_m1), *IL-1b* (Mm00434228_m1) and *CXCL1* (Mm0433859_m1), TNFα (Mm00443258_m1), *SAA* (Mm04208126_mH) supplied by Applied Biosystems were used to assess gene expression in liver and ileum using 10 µl Taqman Fast Universal PCR 2x mastermix No AmpErase UNG (Applied Biosystems, UK) according to the manufacturer's instructions. SYBR real-time PCR analysis of *serpinA3N* was performed using Superarray Bioscience Corporation SYBR green master mix and specific primer pairs for *serpinA3N* (PPM05779F) (Qiagen, UK), according to the manufacturer's instructions. Real-time PCR assays were performed using the ABI-7500Fast (Applied Biosystems, UK). A two-step cycling programme of 95°C 20 seconds, then 40 cycles of 95°C for 3 sec and 60°C for 30 sec was used for Taqman assays and an initial step of 10 minutes at 95°C to activate the HotStart DNA polymerase, followed by 40 cycles of 15 seconds at 95°C and 1 minute at 60°C for SYBR assays. The threshold cycle number (*C*_t_) was measured using the ABI7500Fast associated software (Applied Biosystems).

For assessment of gene expression in gastrocnemius muscle TaqMan probes were designed from the Universal ProbeLibrary, ProbeFinder version 2.45 for mouse (Universal ProbeLibrary, Roche). Primer sequences were; *GAPDH* forward 5′-CCTTGAGATCAACACGTACCAG-3′, reverse; 5′-CGCCTGTACACTCCACCAC-3′; *TNF-α* forward 5′-CTGTAGCCCACGTCGTAGC-3′, reverse 5′-TTGAGATCCATGCCGTTG-3′; *IL-6* forward 5′-GCTACCAAACTGGATATAATCAGGA- 3′, reverse 5′-CCAGGTAGCTATGGTACTCCAGAA-3′; *IL-1β* forward 5′- TGTAATGAAAGACGGCACACC-3′, reverse 5′- TCTTCTTTGGGTATTGCTTGG-3′; and were purchased from Sigma-Aldrich (Gillingham, Dorset, UK), The reactions were carried out in using 10 µl LightCycler 480 Probes MasterMix according to the manufacturer's instructions. Real time PCR assays were performed using the LightCycler 480 system (Roche Diagnostics, Mannheim, Germany). The cycling programme consisted of a pre-incubation of 10 min at 95°C followed by 45 cycles consisting of a 10 seconds at 95°C, and 30 seconds at 60°C. The *C*_t_ number was measured using the Lightcycler 480 system version 5 software (Roche Diagnostics, Mannheim, Germany).

Transcript levels relative to the reference gene, *GAPDH* in muscle and ileum, and *B2M* in liver and WAT were calculated (Δ*C*_t_). Fold expression changes between experimental groups relative to *GAPDH* or *B2M* were calculated from the ΔΔ*C*_t_ values (n = 6–8). As these values are ratios there are no standard errors.

### Two-dimensional gel electrophoresis (2-DE)

The 2-DE was performed essentially as detailed previously with some modifications [24]. Bio-Rad, 11 cm, immobilized pH gradient (IPG) strips (pH 3–10) were used for the separation of plasma proteins in the first dimension. Strips were rehydrated in rehydration buffer (7 M urea; 2 M thiourea; 4% w/v CHAPS; 2% w/v Biolyte; and 50 mM DTT) containing 200 µg of protein sample in a Bio-Rad IEF cell and then focused.

After the first dimension IPG strips were incubated in fresh equilibration buffer (6 M urea; 2% w/v SDS; 0.375 M Tris-HCl, pH 8.8; 20% v/v glycerol; and 130 mM DTT) for 10–15 min at room temperature before transfer to a second equilibration buffer (6 M urea; 2% w/v SDS; 0.375 M Tris-HCl, pH 8.8; 20% v/v glycerol; and 135 mM iodoacetamide) for 10–15 min at room temperature. The strip was then applied to the top of a precast Criterion XT Bis-Tris 3–12% IPG+1 well gel cassette and 5 µl of All Blue Precision Protein Standards (Bio-Rad) was loaded in the reference well. Gels were run at 200 V until the bromophenol blue had reached the bottom of the gel. The gels were fixed and stained with Coomassie Blue (n = 5).

### Identification of mouse plasma proteins

The 2-DE gels were analysed using Progenisis Samespots software (Nonlinear Dynamics Ltd, UK). Spots which showed differences in normalised average volume with *P*<0.05 by Anova in HF *vs*. LF were cut from SDS-PAGE gels. Gel plugs were trypsinized using the MassPrep Station (Waters, Micromass, UK) protocol. Spot identification was carried out by LC/MS/MS as described previously [24] using an ‘Ultimate’ nanoLC system (LC Packings, UK) and a Q-Trap (Applied Biosystems/MDS Sciex, UK) triple quadrupole mass spectrometer fitted with a nanospray ion source. The total ion current (TIC) data were submitted for database searching using the MASCOT search engine (Matrix Science, UK) using the MSDB database.

### Western blot analysis

Mouse plasma samples were separated by electrophoresis as reported previously [24]. Bands on the blots from LF fed animals were considered as equivalent to 1 and those from HF animals scored accordingly. Mouse plasma samples containing 10 µg of total plasma protein were separated by electrophoresis using precast Criterion XT Bis-Tris Gels (Bio-Rad). Following electrophoresis the gels were either stained using Simply Blue Safe Stain (Invitrogen, UK) to verify protein loading or the proteins were transferred onto PVDF membranes [24] and protein transfer was confirmed by Ponceau red staining. The membranes were then probed with the following primary antibodies; haptoglobin antibody (ab8968) (AbCam, Cambridge, UK), ApoA-IV(N20) antibody (Santa Cruz Biotechnology, Dallas, US) and mouse serpinA3N antibody (R&D Systems, Abingdon, UK). Following incubations with the primary antibodies the membranes were incubated with secondary antibodies conjugated to HRP (AbCam). The blots were then developed using chemiluminesent detection reagent (Merck Millipore, Feltham, UK) and imaged using the FujiFilm LAS-3000 system. The resulting gel and blot bands were then analysed using TotalLab software (Nonlinear Dynamics, UK). Bands on the blots from LF fed animals were considered as equivalent to 1 and those from HF animals scored accordingly (n = 6–8).

### Measurement of plasma hormones

Measurements were carried out using Luminex's xMAP Technology and Milliplex MAP mouse serum adipokine panel for insulin and leptin and Milliplex MAP mouse cytokine/chemokine panel for IL-1β, IL-6 and TNFα (Merck Millipore, Feltham, UK). All measurements were carried out on terminal blood samples from fed animals (n = 6–8).

### Measurement of plasma lipids

Analyses were carried out on terminal blood samples from fed mice (n = 6–8) using a Thermo Konelab 30 clinical analyser and kits for HDL and LDL cholesterol and (Microgenics GmbH Passau, Germany) and non-esterified fatty acid (NEFA) (Alpha Laboratories Eastleigh, UK).

### Statistical Analysis

Data are represented as mean ± SEM and were analyzed using GenStat (GenStat, 10th Edition, VSN International Ltd, Oxford). In the case of experiments testing the influence of a single factor a one-way ANOVA was performed. Where two factors were involved data were analysed by two-way ANOVA. *P*<0.05 was considered statistically significant.

## Results

### Body Weight and Composition

**Figure 1:**
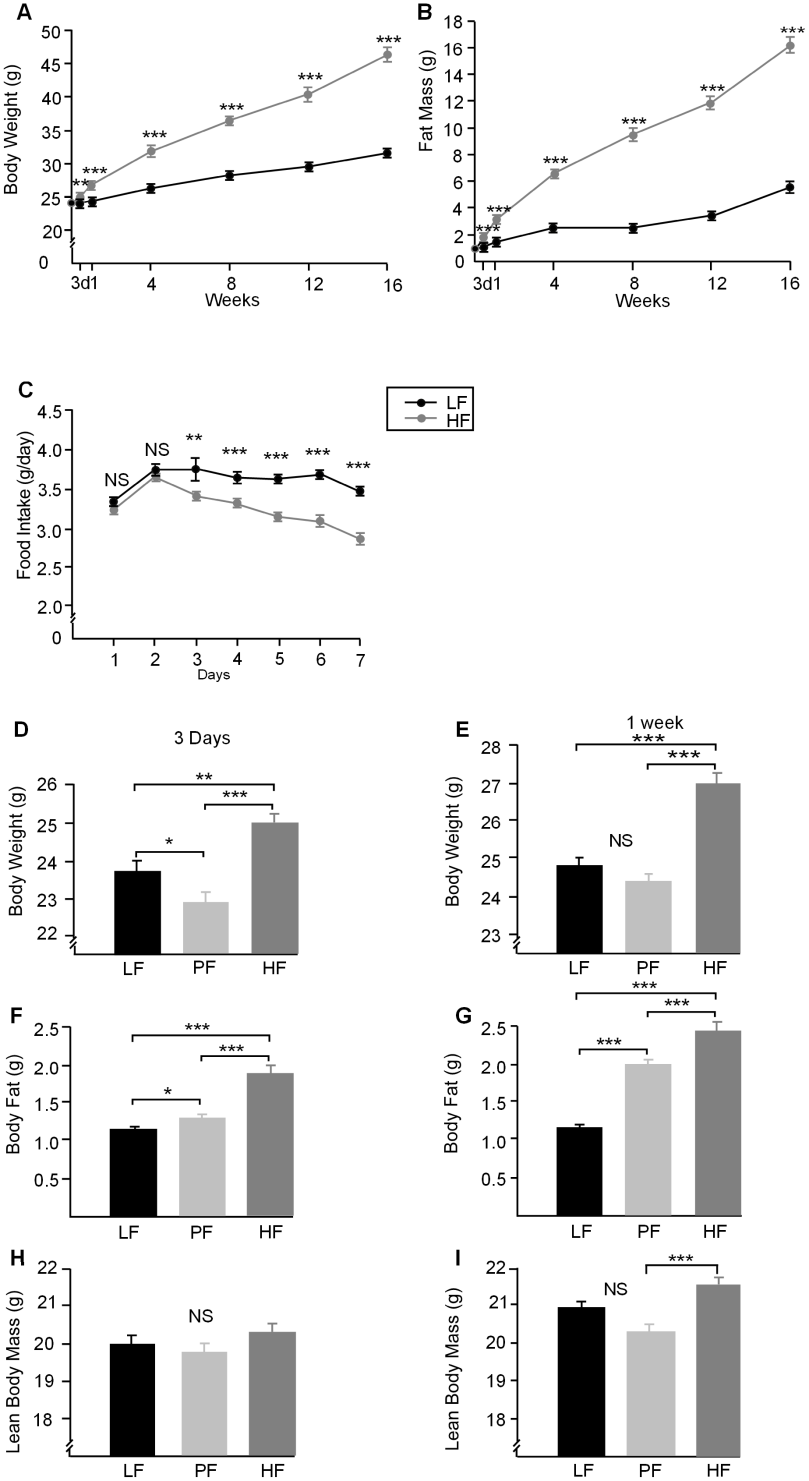
**A.** Body weight of the HF fed mice differed significantly from those of the LF fed mice from 3 days of feeding (*P*<0.01) and (*P*<0.001) onwards. **B.** The increase in body weight seen in A is mostly fat mass which was increased in the HF mice from 3 days onwards (*P*<0.001). **C.** Mean food intake of mice receiving either a LF or HF diet (grams per day). The intake of the HF fed mice was decreased at 3 days on diet (*P*<0.01) and (*P*<0.001) thereafter (n = 6–8). **D.** PF animals showed a decrease in body weight after 3 days on diet compared to LF (*P*<0.05 and HF fed animals (*P*<0.001). **E.** PF animals had a similar body weight to LF fed mice after 1 week on diet. Both LF and PF mice had lower body weight than the HF mice (*P*<0.001). **F.** Body fat measured by MRI after 3 days of diet was increased in PF (*P*<0.05) and HF mice (*P*<0.001) compared to LF mice despite the lower body weight seen in PF mice in C. **G.** Body fat after 1 week on diet was higher in both PF (*P*<0.001) and HF fed mice (*P*<0.001) compared to LF and was also higher in HF (*P*<0.001) compared to PF mice. **H.** Lean body mass after 3 days on diet was similar in all mice LF, PF and HF indicating that all weight changes were due to changes in adiposity. **I.** Lean mass after 1 week on diet was only increased in HF fed mice compared to LF and PF (*P*<0.001) (n = 6–8).

Body weight and adiposity of mice fed a HF diet were significantly increased after 3 days on diet and both continued to increase linearly with time compared to the LF fed mice (Fig 1*A*–*B*). Food intake dropped significantly in HF fed mice at day 3 on the diet and this lower level was maintained (Fig 1*C*). PF mice showed a drop in body weight after 3 days compared to both LF and HF diets (Fig 1*D*), after 1 week body weight was similar in the LF and PF groups which were both lower that the HF fed mice (Fig 1*E*). Despite a lower body weight after 3 days on the diet the PF mice have increased adiposity compared to the LF fed mice (Fig 1*F*). By 1 week on diet the PF mice have further increased adiposity compared with the LF mice (Fig 1*G*) and have a lower lean mass compared to HF fed animals (Fig 1*I*). There were no difference in lean mass at 3 days or 1 week between LF and HF fed mice (Fig 1H & *I*).

### Liver and Circulating Lipids

**Figure 2:**
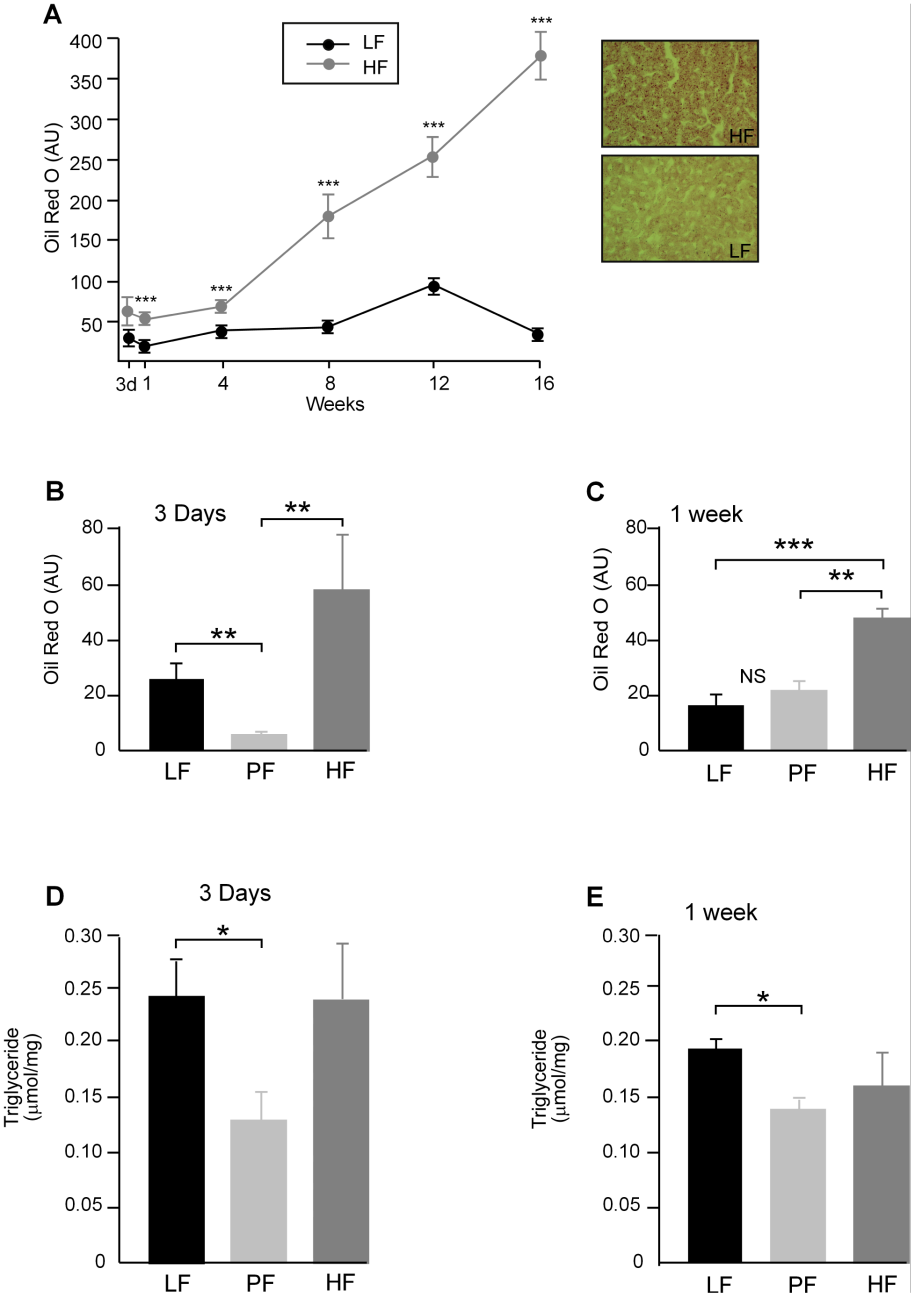
**A, B &C.** Liver lipid measured by image analysis of oil red O in arbitrary units (AU). **A.** staining increased linearly with time on diet in HF fed mice from 1 week (*P*<0.001) onwards. Representative images of oil red O stained sections for HF and LF fed mice after 8 weeks on diet. Mag X 100. **B.** Liver lipid in PF mice was reduced compared to both LF and HF mice after 3 days (*P*<0.01). There was no difference in liver lipid between LF and HF fed mice. **C.** After 1 week on diet there was no difference in liver lipid between LF and PF mice but HF mice had more lipid than LF (*P*<0.001) and PF (*P*<0.01) mice (n = 6–8). **D & E.** Liver (TAG), measured by Kone analysis. **D.** LF and HF levels are not significantly different while PF mice show a lower level of TAG compared to LF fed mice (*P*<0.05) after 3 days on diet. **E.** LF and HF levels are not significantly different while PF mice show a lower level of TAG compared to LF fed mice (*P*<0.05) after 1 week on diet.

**Figure 3:**
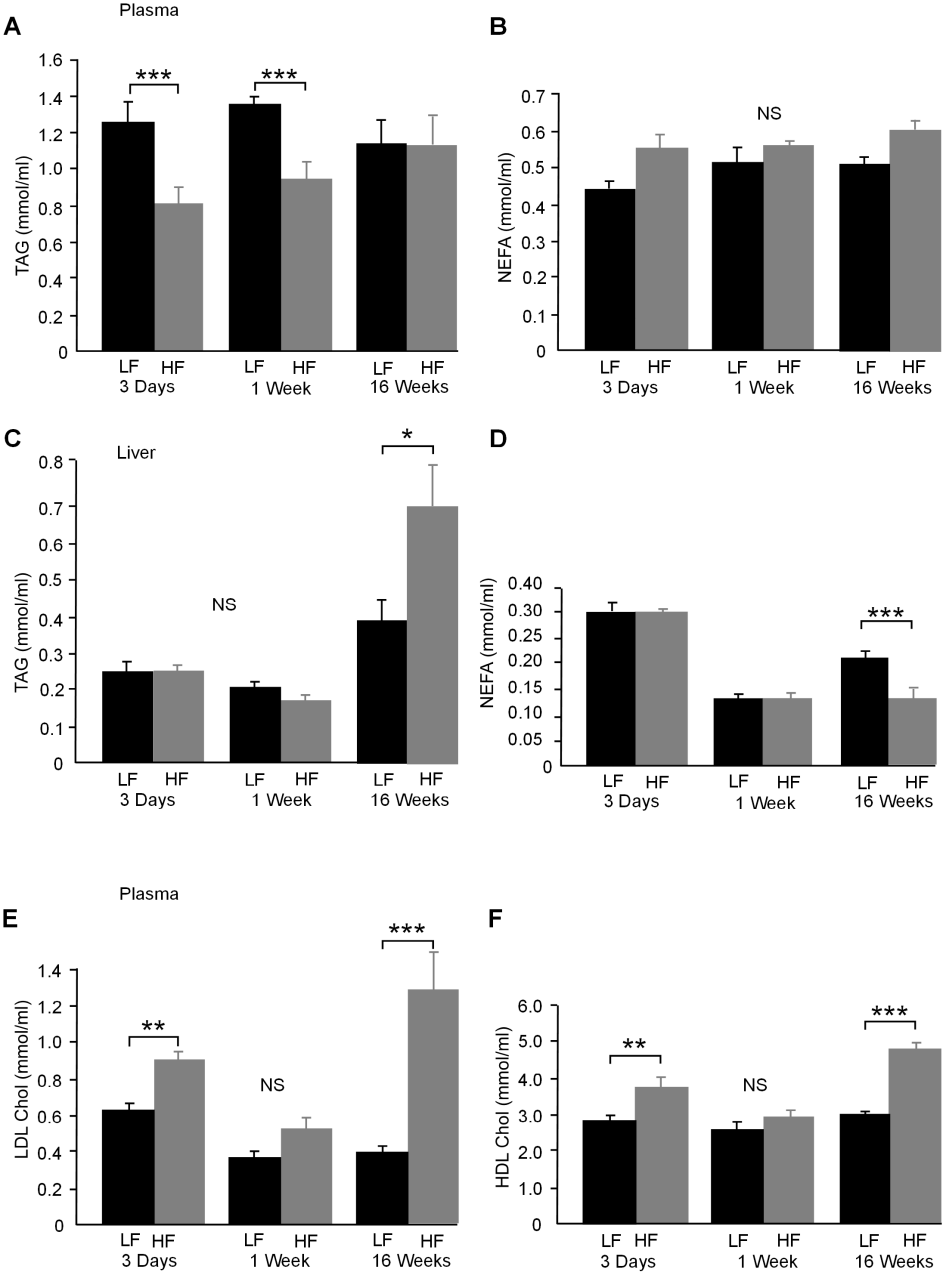
**A.** Plasma triacylglycerol (TAG) was significantly lower in HF fed mice at both 3 days and 1 week on diet (*P*<0.001), but did not differ at 16 weeks on diet. **B.** Plasma non esterified fatty acids (NEFA)/free fatty acids were unchanged by a HF diet at all times tested. **C.** Liver TAG levels were unchanged in the HF fed mice at 3 days and 1 week and significantly higher after 16 weeks on HF diet (*P*<0.05). **D.** Liver NEFA levels were unchanged at 3 days and 1 week on HF diet but were significantly lower after 16 weeks on HF diet (*P*<0.001) **E.** Plasma low-density lipoprotein (LDL) cholesterol was elevated in HF fed mice after 3 days (*P*<0.01) and 16 weeks (*P*<0.001) on diet, but was not different from LF values after 1 week. **F.** Plasma high-density lipoprotein (HDL) cholesterol followed the same pattern as LDL cholesterol and was higher in HF fed mice at 3 days (*P*<0.01) and 16 weeks (*P*<0.001) on diet, but was not different from LF after 1 week of diet (n = 6–8).

Liver lipid, measured by oil red O, increased linearly (Fig 2*A*) but was not significantly increased after 3 days on HF diet. Liver lipid measured by oil red O was lower in the PF mice compared to the LF after 3 days (Fig 2*B*). By 1 week on diet there was no difference in liver lipid between LF and PF animals but HF animals had higher levels of liver lipid, measured by oil red O, than PF or LF mice (Fig 2*C*). Liver triacylglycerol (TAG) levels, measured by Kone analysis, showed a reduction in liver TAG after 3 days in the PF mice and no difference between LF and HF mice at this time (Fig 2*D*). Liver TAG, measured by Kone analysis, after 1 week showed lower levels in PF mice compared to HF mice but no difference between LF and HF mice (2*E*). Plasma triacylglycerol (TAG) levels were significantly lower in the HF fed mice at 3 days and 1 week and was unchanged after 16 weeks on HF diet (Fig 3*A*). Plasma non esterified fatty acids (NEFA) levels were unchanged at all times tested (3*B*). Liver TAG levels were unchanged in the HF fed mice at 3 days and 1 week and significantly higher after 16 weeks on HF diet (Fig 3*C*). Liver NEFA levels were unchanged at 3 days and 1 week on HF diet but were significantly lower after 16 weeks on HF diet (Fig 3*D*). Serum LDL and HDL cholesterol were elevated in HF fed mice at 3 days and again at 16 weeks but were unchanged at 1 week on HF diet (Fig 3*E.*&*F*).

### Glucose tolerance

**Figure 4:**
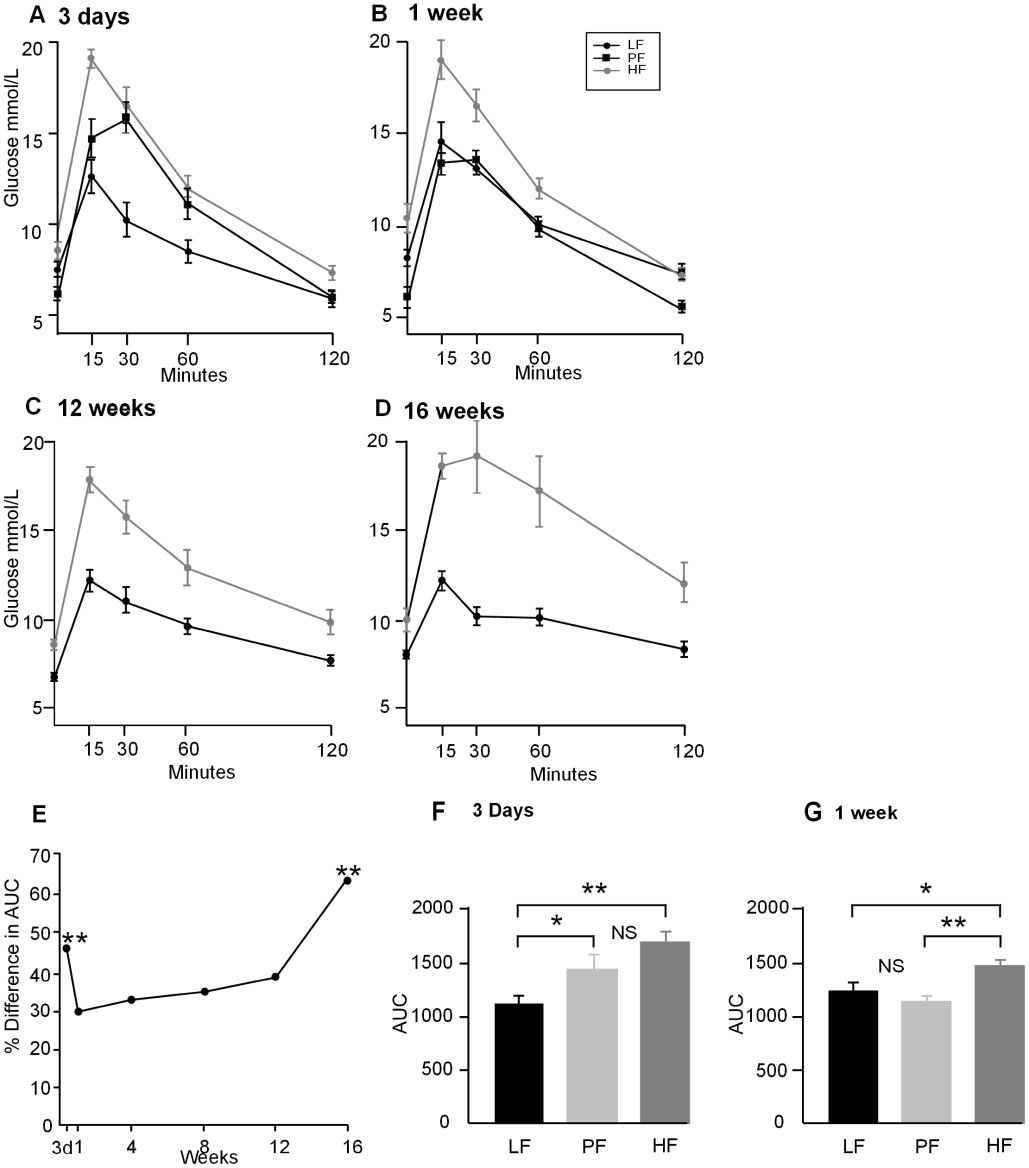
**A–D.** Intraperitoneal glucose tolerance tests (IPGTT) in LF fed mice •, HF fed mice • and PF fed mice ▪, fed the HF diet restricted to the caloric intake of the LF fed mice, after 3 days, 1, 12 and 16 weeks after the start of diet. IPGTT was carried out as a non-recovery procedure as the effect of fasting and glucose administration can alter gene expression for some time afterwards. (n = 6–8). **E.** Comparison of the % difference in total AUC between LF and HF mice over time using a two-way ANOVA showed that when time points 3 days and 16 weeks were included that diet and time had a significant effect on glucose tolerance (F_1,58_ = 94.56, *P*<0.01) and (F_4,58_ = 11.22, *P*<0.01) respectively and that there was an interaction between diet and time (F_4,58_ = 2.83, *P*<0.05). However, when these time points were omitted there was no effect of time or diet indicating that there was no change in AUC between 1 and 12 weeks. One way ANOVA of the difference between LF and HF total AUC revealed that is was higher at 3 days (*P*<0.01) and 16 weeks (*P*<0.01) compared to the other time points tested and that there was no change in the % difference in total AUC between 1, 4, 8 and 12 weeks on diet. **F.** A comparison of total AUC in LF, PF and HF fed mice after 3 days on diet shows the PF (*P*<0.05) and HF (*P*<0.01) mice have higher total AUC compared to LF mice but that the total AUC for PF and HF was not significantly different (NS). **G.** A comparison of total AUC in LF, PF and HF fed mice after 1 week on diet shows a different pattern with no difference in total AUC between LF and PF mice (NS). The total AUC for HF mice is significantly higher than PF (*P*<0.01) and LF mice (*P*<0.05) (n = 6–8).

Total AUC was increased in HF fed mice at all the time points tested (Fig 4*A–D*). When the % difference in AUC between LF and HF fed mice was compared for all time points, tested using a two-way ANOVA for diet and time interaction, there were significant effects of both diet (*P*<0.01) and time (*P*<0.01) and an interaction between diet and time (*P*<0.05). However, when the 3 day and 16 week data were excluded from the analysis there was no significant difference between total AUC at the individual time points of 1, 4, 8 and 12 weeks on the HF diet showing that glucose intolerance remained stable between these times (Fig 4*E*). Glucose tolerance after 3 days on diet showed that both PF and HF mice had a similar AUC which was higher than the LF mice (Fig 4*F*). By 1 week on diet the PF mice had similar AUC to the LF mice and HF mice had a higher AUC than both (Fig 4*G*).

### Circulating leptin, insulin and basal glucose levels

**Figure 5:**
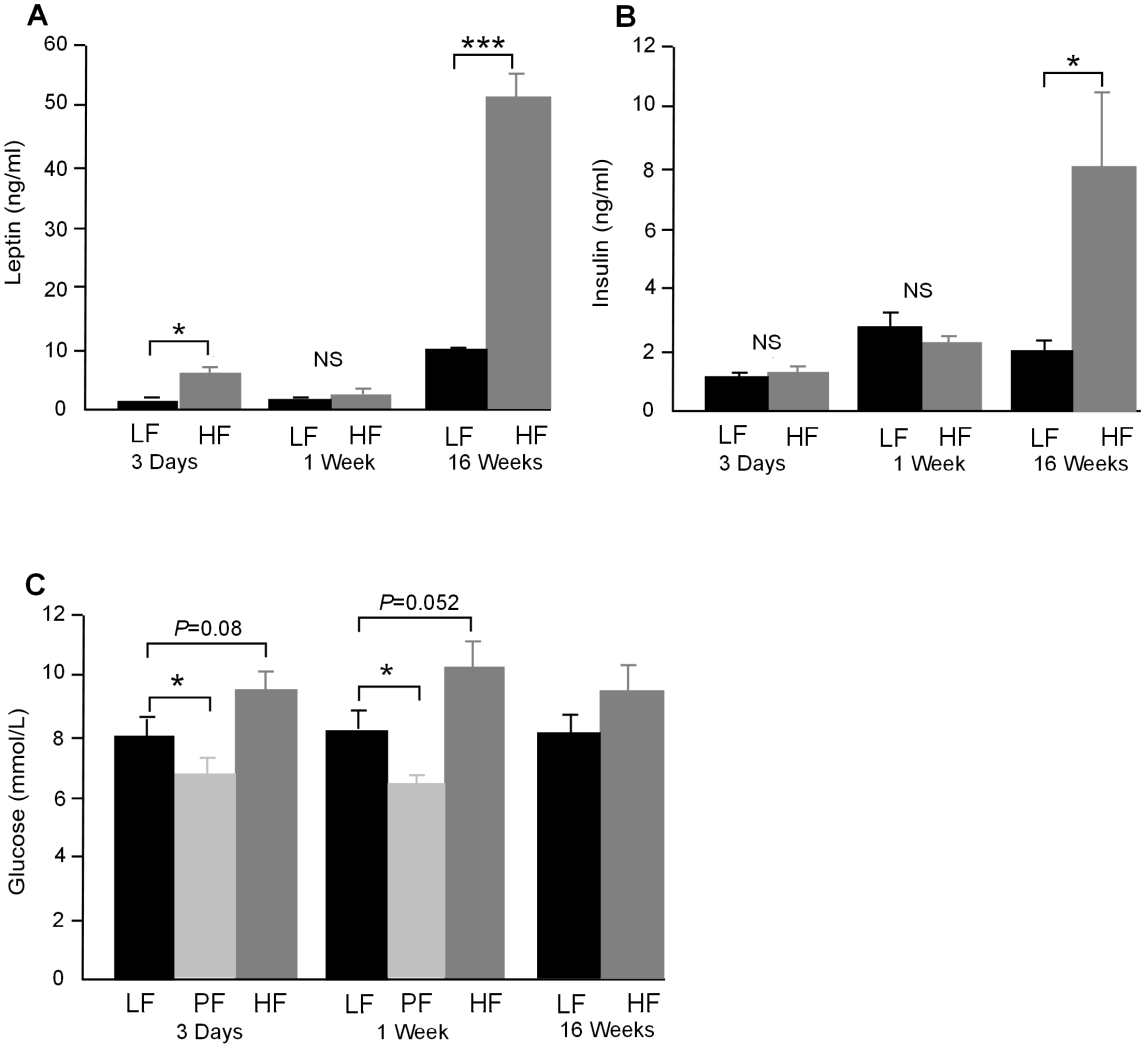
**A.** Plasma leptin levels were significantly higher in HF fed mice after 3 days on diet (*P*<0.05), did not differ after 1 week on diet (NS) and increased greatly after 16 weeks on HF diet (*P*<0.001). **B.** Plasma insulin levels were not different in HF mice compared to LF fed mice after 3 days or 1 week on diet (NS) but were increased after 16 weeks (*P*<0.05). **C.** Fasted glucose levels approached statistical significance for HF *vs.* LF after 3 days and 1 week on diet (*P* = 0.08 and *P* = 0.052 respectively) but was not different at 16 weeks. Fasted glucose in PF mice was significantly lower than LF mice at both 3 days and 1 week of feeding (*P*<0.05) (n = 6–8).

Circulating leptin levels were increased after both 3 days (*P*<0.05) and 16 weeks (*P*<0.001) on the HF diet but not after 1 week (Fig 5*A*). Insulin levels were unchanged at 3 days and 1 week on HF diet but were significantly increased after 16 weeks (*P*<0.05) (Fig 5*B*). There was a large variability in insulin levels measured as animals in this study were not fasted. Basal fasted glucose levels in HF mice compared to LF approached significance after both 3 days (*P* = 0.08) and 1 week (*P* = 0.052) on diet but were not different after 16 weeks. Fasted glucose levels in PF mice were significantly lower than LF and HF fed mice (*P*<0.05) at 3 days and 1 week on diet which may be indicative of a more prolonged fasting period than that imposed on the other mice (Fig 5*C*).

### Plasma proteomics and Western blotting

**Figure 6:**
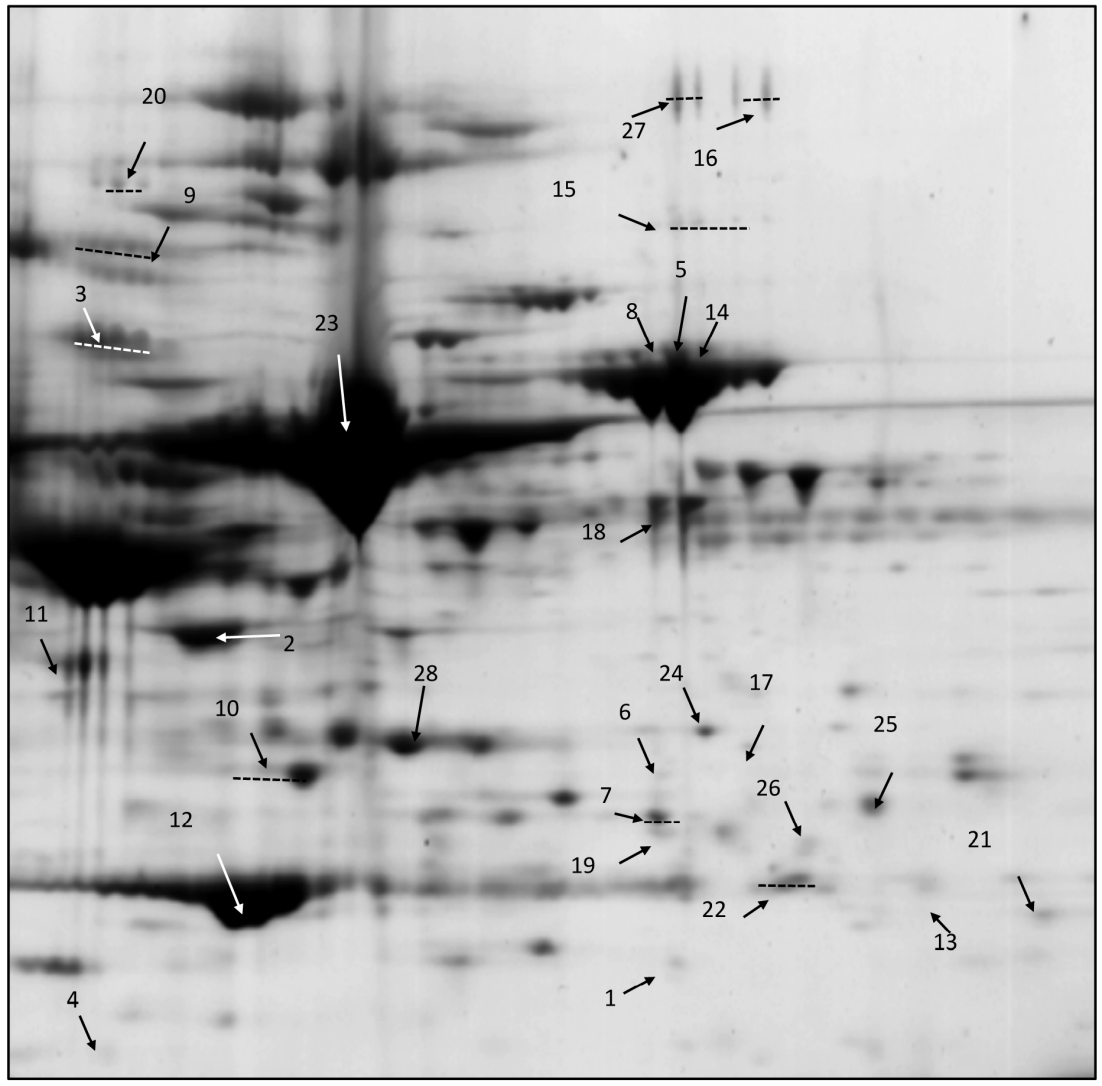
Representative 2D Coomassie stained gel of mouse plasma after 3 days on the HF diet. Bio-Rad, 11 cm, immobilized pH gradient (IPG) strips (pH 3–10) were used for the separation of plasma proteins in the first dimension. After the first dimension the IPG strip was applied to the top of a precast Criterion XT Bis-Tris 3–12% IPG+ 1 well gel cassette and 5 µl of All Blue Precision Protein Standards (Bio-Rad) were loaded in the reference well. The gels were fixed and stained with Coomassie Blue. Numbered spots indicate those with significantly different average normalised volumes (*P*<0.05) (n = 5) in HF compared to LF mice. Proteins were identified by LC/MS/MS. Spots inside dotted lines have been identified as the same protein. See Table 1 for protein identification.

**Figure 7:**
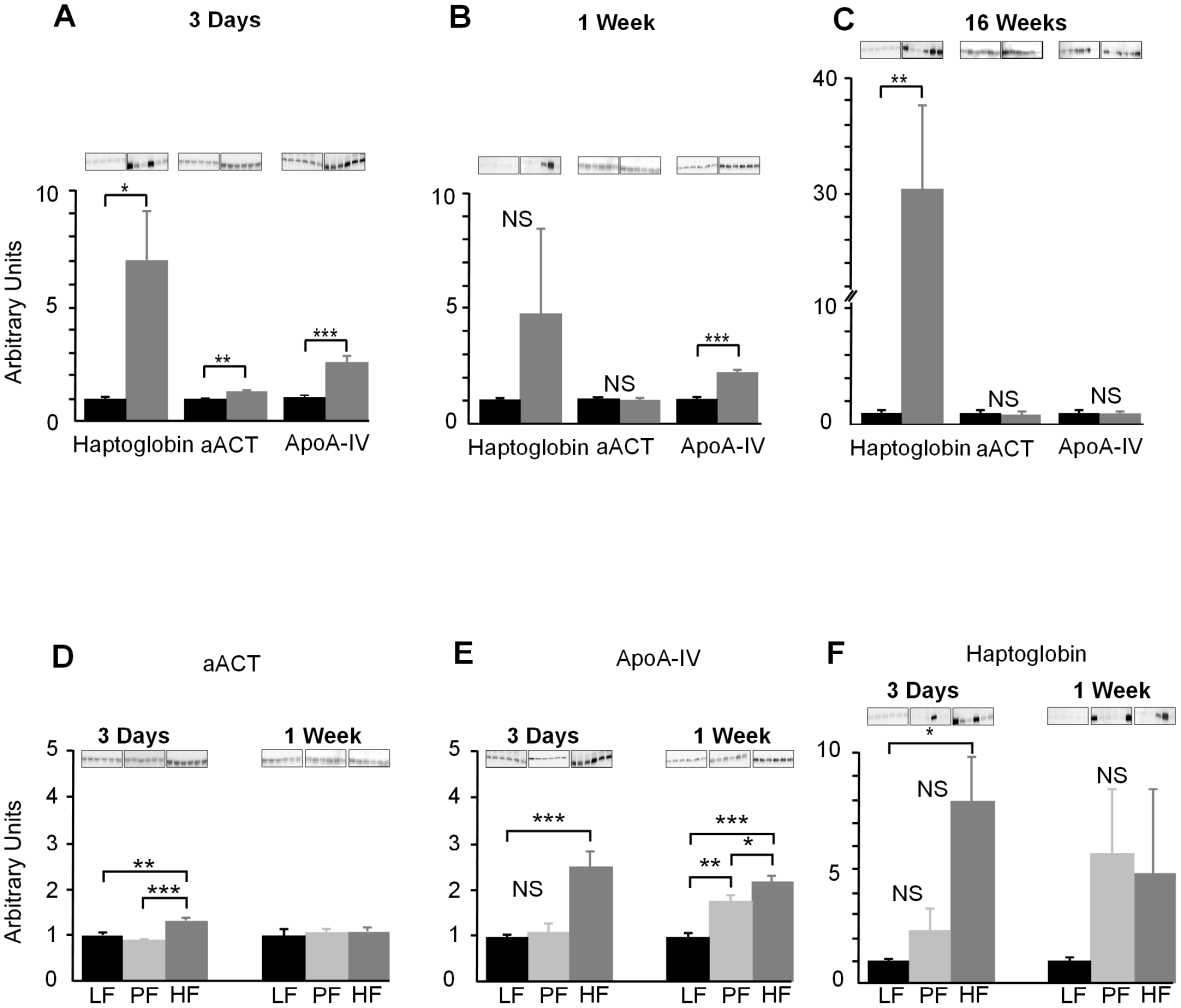
**A.** Plasma protein levels of haptoglobin (*P*<0.05), alpha-1-antichymotrypsin (aACT) (*P*<0.01) and ApoA-IV (*P*<0.001) were all significantly higher in HF compared to LF after 3 days on the diet. **B.** After 1 week on the diet plasma protein levels of ApoA-IV were significantly greater in HF fed mice compared with LF (*P*<0.01). There was no significant difference in haptoglobin or aACT protein levels between mice on HF *vs.* LF diets after 1 week. **C.** After 16 weeks on the diet there was significantly greater level of haptoglobin protein in plasma from HF compared to LF (*P*<0.01), there was no significant difference in the levels of ApoA-IV or aACT in HF compared to LF (n = 6–8). **D.** Alpha-1-antichymotrypsin (aACT) plasma protein levels were significantly greater in HF compared to LF (*P*<0.01) and PF (*P*<0.001) at 3 days. There were no significant differences in aACT plasma protein levels after 1 week. **E.** ApoA-IV plasma protein was significantly increased in HF compared to LF at 3 days (*P*<0.001). At 1 week ApoA-IV plasma protein levels were higher in HF compared to both LF (*P*<0.001) and PF (*P*<0.05). The amount of ApoA-IV protein was also greater in plasma from PF mice compared to LF (*P*<0.01) after 1 week. **F.** Levels of haptoglobin protein in plasma from HF mice were significantly greater at 3 days compared to LF (*P*<0.05). There was no significant difference in plasma protein levels of haptoglobin between the different diets after 1 week (n = 6–8).

Analysis of 2-DE revealed a total of 28 soluble proteins, from a total of 247, that were significantly changed between the LF and HF fed mice after 3 days on diet (Fig 6). These were analysed by LC-MS/MS and the majority were classified as acute phase proteins (Table 1). To extend these findings, two typical acute phase proteins; haptoglobin and alpha-1-antichymotrypsin (aACT) and one atypical acute phase protein, ApoA-IV were measured by Western blotting to confirm their up-regulation in the HF-fed animals. After 3 days on diet (Fig 7*A*), all proteins were increased compared with the LF control. After 1 week on the diet the levels of haptoglobin and aACT had dropped but ApoA-IV remained elevated (Fig 7*B*). After 16 weeks on the HF diet only haptoglobin was elevated (Fig 7*C*). In plasma from PF mice levels of haptoglobin and aACT were comparable with mice on a LF diet at 3 days and 1 week, while levels in the HF animals were significantly elevated after 3 days on diet. After 1 week on the diets only the ApoA-IV showed an up-regulation in both the PF and HF groups compared to the LF (Fig 7*D–F*).

**Table 1:** Protein identification by LC/MS/MS of spots in 2DE gels of 3 day mice which were significantly different in averaged normalised volume in HF compared to LF fed mice (n = 5).

Spot #	Accession No.	Protein Name	Peptides Matched	Anova (*P*)	Fold Change	pI	Mr (kDa)	Average Normalised Volumes	
								High-fat	Low-fat
1	S27001	alpha-2-macroglobulin - mouse	7	0.002	2.1	7.2	19.1	2200.356	1072.151
2	Q9DBN0_MOUSE	apolipoprotein A-IV*	22	0.002	1.4	5.5	41.7	1.487e+005	1.088e+005
3	C3HUQ8VC41_MOUSE	serpina1d protein*	19	0.004	1.4	5.1	84.2	1.543e+005	1.118e+005
4	VBMS	transthyretin precursor-mouse*	1	0.004	2.1	5.1	15.5	394.133	820.807
5	Q3UBW7_MOUSE	transferrin - mouse*	25	0.004	2.1	7.3	80.7	1.255e+004	5958.267
6	S27001	alpha-2-macroglobulin - mouse*	1	0.011	1.8	7.2	31.1	1843.010	1022.351
7	CAH1_MOUSE	carbonic anhydrase 1*	7	0.012	1.6	7.2	28.2	1.888e+004	1.207e+004
8	KQMSPL	plasma kallikrein precursor - mouse*	2	0.007	1.9	7.2	80.7	2.088e+004	1.339e+004
9	Q5I0Y2_MOUSE	leukemia inhibitory factor receptor	22	0.013	1.5	5.5	109.1	4.871e+004	3.286e+004
10	Q3UBS0_MOUSE	Apoe protein.-mouse*	15	0.007	1.2	5.9	31.1	6.968e+004	5.757e+004
11	S20045	MHC class I histocompatibility antigen* Q10-k alpha chain -mouse	4	0.041	1.3	5.0	38.3	3.982e+004	3.041e+004
12	Q3V2G1_MOUSE	apolipoprotein A-I*	4	0.014	1.2	5.6	22.0	6.018e+005	4.839e+005
13	Not identified	not identified		0.015	1.4	8.1	23.9	4229.688	5970.531
14	KQMSPL	plasma kallikrein precursor - mouse*	5	0.007	1.9	7.3	80.7	1.633e+004	8586.978
15	Not identified	not identified		0.017	1.5	7.3	118.8	8759.651	5808.859
16	Q3UBW7_MOUSE	transferrin*	4	0.020	3.3	7.4	192.4	1.406e+004	4311.574
17	Not identified	not identified		0.021	1.8	7.5	33.1	536.483	291.251
18	Q99K47_MOUSE	fibrinogen, alpha*	6	0.023	1.4	7.2	56.2	7.500e+004	5.482e+004
19	1KB5L	antibody desire-1 fab cleaved by papain, chain L - mouse	3	0.023	1.6	7.1	27.4	4550.480	2862.626
20	Q8VC41_MOUSE	serpina1d protein*	6	0.005	2.3	5.2	135.1	3.983e+004	2.380e+004
21	1GTIA	glutathione s-transferase, chain A - mouse*	2	0.033	1.4	8.6	21.8	3341.933	2436.880
22	1NLDL	fab1583, chain L – mouse	5	0.038	1.3	7.7	23.8	3.357e+004	4.209e+004
23	Q3TV03_MOUSE	albumin 1-mouse*	60	0.033	1.2	5.9	63.2	3.025e+006	3.697e+006
24	A24558	complement C4 precursor - mouse	2	0.042	1.4	7.5	33.8	9677.175	6917.239
25	Not identified	not identified		0.044	1.2	7.9	28.2	1.809e+004	1.525e+004
26	Q3TV03_MOUSE	albumin 1-mouse*	7	0.041	1.4	7.7	26.9	2024.326	1474.452
27	Q3UBW7_MOUSE	transferrin*	11	0.044	1.8	7.2	184.8	5.516e+004	3.107e+004
28	Q6PEM2_MOUSE	pregnancy zone protein	9	0.049	1.2	6.2	33.0	6.600e+004	7.925e+004

Proteins identified as acute phase are marked*.

### Inflammatory markers in the plasma

**Figure 8:**
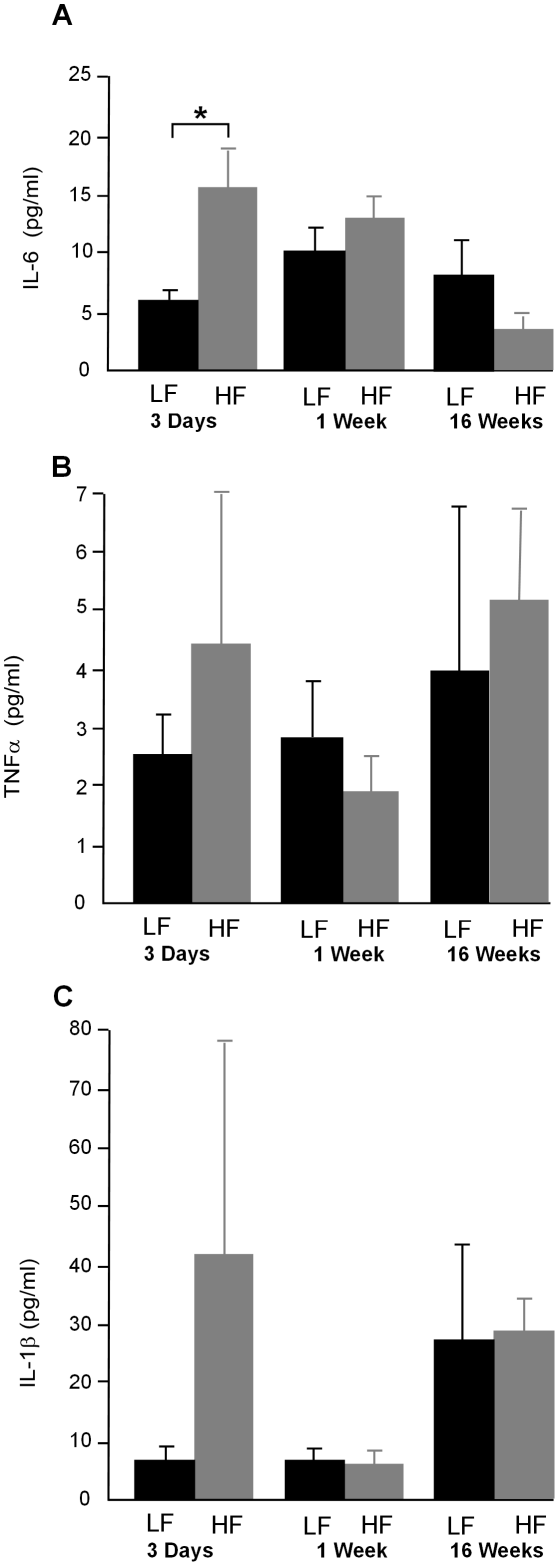
**A.** Plasma levels of IL-6 were increased after 3 days on a HF diet (*P*<0.05) but were unchanged after 1 week and 16 weeks on diet. **B.** Plasma levels of TNFα were not significantly different at any of the time points tested but individual measurements were variable. **C.** As with TNFα plasma levels of IL -1β were not significantly different at any of the time points tested and individual measurements were variable particularly in HF fed mice after 3 days on diet (n = 6–8).

Plasma levels of IL-6 were increased in HF fed mice after 3 days on diet (*P*<0.05) (Fig 8*A*). Plasma levels of TNFα and IL-1β were not significantly different between HF and LF mice at any of the time points measured but showed large variability in circulating levels particularly in the HF fed mice at day 3 (Fig 8*B*&*C*).

### Acute phase protein gene expression in liver and ileum

**Figure 9:**
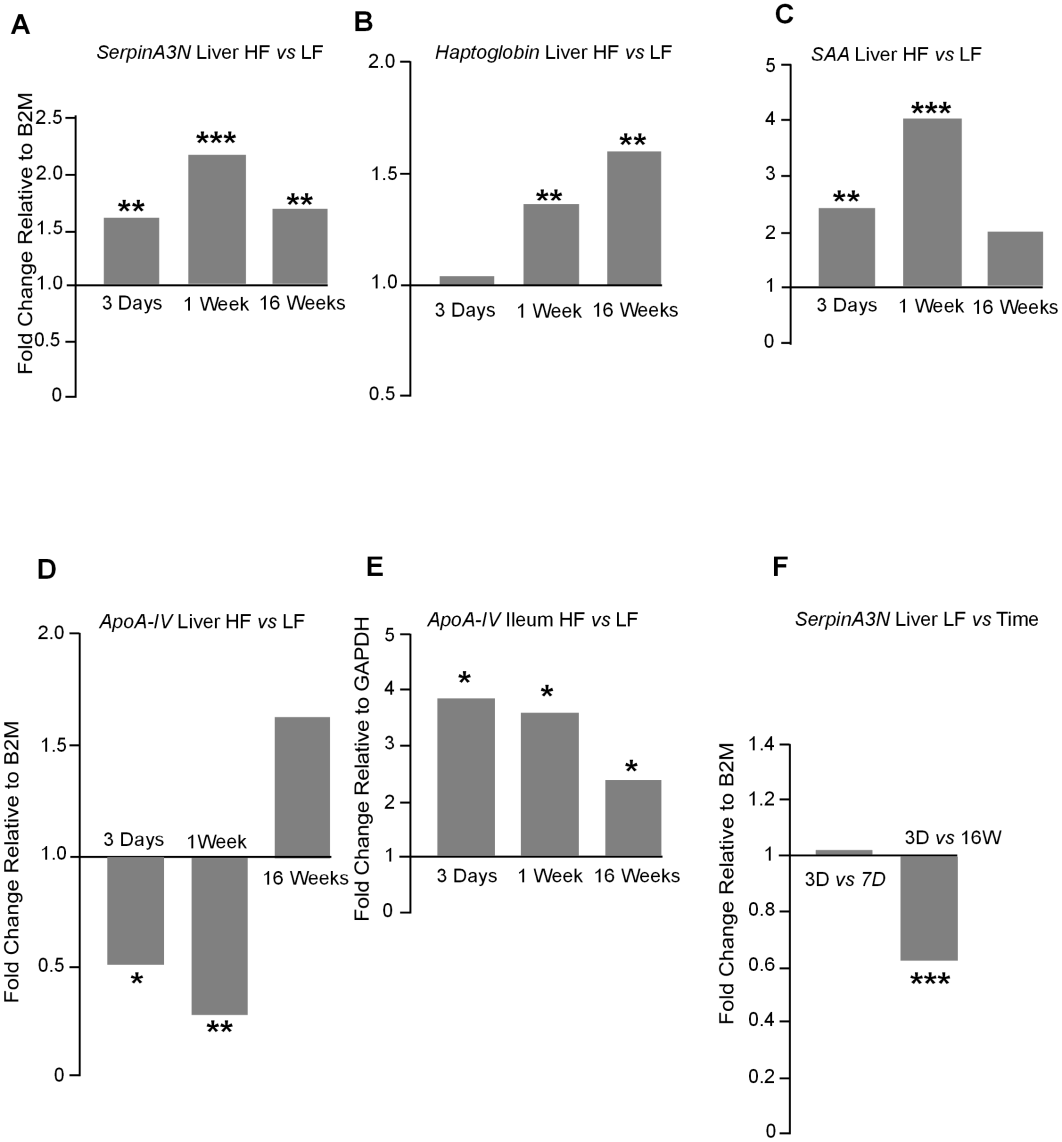
**A.** *SerpinA3N* gene expression in the liver was significantly up-regulated in a HF diet at all times tested 3 days (*P*<0.01), 1 week (*P*<0.001) and 16 weeks (*P*<0.01). **B.** *Haptoglobin* gene expression was unchanged in HF fed mice liver after 3 days on diet but was increased after 1 week (*P*<0.01) and 16 weeks (*P*<0.01). **C.** *SAA* gene expression was significantly higher on a HF diet after 3 days (*P*<0.01) and 1 week (*P*<0.001) but was not different after 16 weeks. **D.** *ApoA-IV* gene expression was significantly down-regulated in the liver of HF fed mice after 3 days (*P*<0.05) and 1 week (*P*<0.01) on diet but was unchanged after 16 weeks on diet. **E.** In comparison *ApoA-IV* gene expression was significantly up-regulated in the ileum of HF fed mice after 3 days, 1 week and 16 weeks on diet (*P*<0.05). **F.** *SerpinA3N* gene expression in the liver was significantly down-regulated with time on a LF diet between 3 days and 16 weeks (*P*<0.001). Units are fold expression changes between experimental groups relative to *B2M* and calculated from the ΔΔ*C*_t_ values (n = 6–8).

The expression of acute phase protein genes in the liver on the HF diet was elevated for *serpinA3N* (the gene encoding aACT) at 3 days, 1 week and 16 weeks, *haptoglobin* at 1 week and 16 weeks and serum amyloid A at 3 days and 1 week, however, the level of up-regulation varied (Fig 9*A*–*C*). In contrast to circulating levels, gene expression of *ApoA-IV* in the liver was significantly down-regulated in HF compared to LF animals at day 3 and 1 week on the HF diet and were not different at week 16 (Fig 9*D*). However, *ApoA-IV* gene expression in the ileum was significantly up-regulated at all three times tested (Fig 9*E*). *SerpinA3N* gene expression showed variation with time on diet with down-regulation at 16 weeks compared with 3 days on LF diet (Fig 9*F*).

### Macrophage area and inflammatory marker gene expression in liver

**Figure 10:**
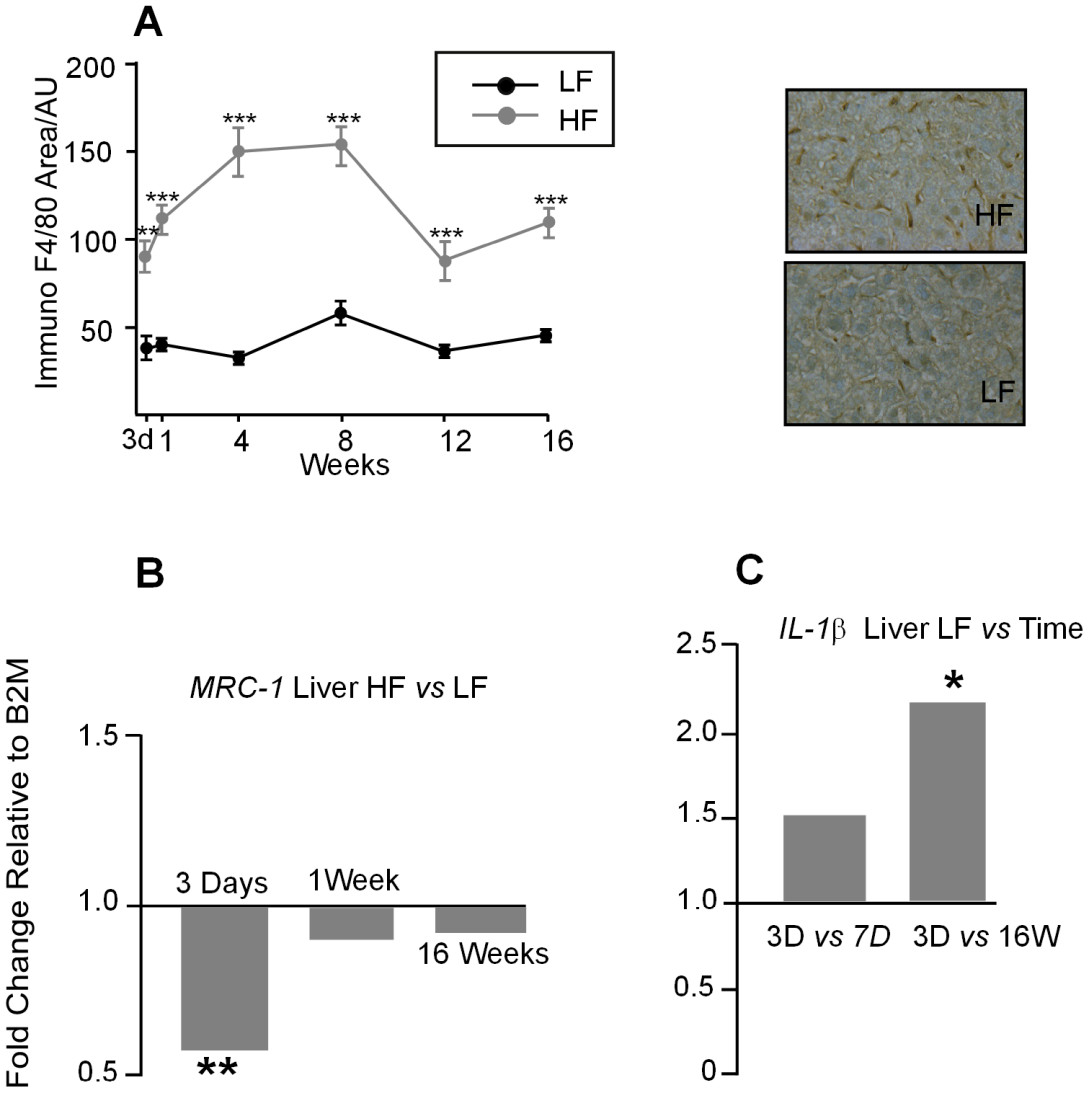
**A.** The area of F4/80 staining in the liver measured as arbitrary units (AU) was significantly increased in HF fed mice at all the time points tested (*P*<0.01) at 3 days and (*P*<0.001) at all other time points. Values appeared to peak at 4 weeks on the HF diet. Representative images of F4/80 staining (brown) for HF and LF fed mice after 1 week on diet. Mag X 200. **B.** *MRC-1* gene expression was significantly decreased after 3 days on a HF diet (*P*<0.001) but was unchanged after 1 week and 16 weeks. None of the other genes tested *Il-1β, IL-6, TNFα, CXCL1, F4/80, MRC-1, ARG* and *ITgax* showed any change with time on a HF diet. *IL-10* expression was undetectable (data not shown) (n = 6-8). **C.** *IL-1β* gene expression in the liver is up-regulated between 3 days and 16 weeks on a LF diet (*P*<0.05). Units are fold expression changes between experimental groups relative to *B2M* and calculated from the ΔΔ*C*_t_ values (n = 6–8).

The area occupied by liver Kupffer cells, measured by F4/80 immunohistochemistry, was increased in the HF fed mice compared to the LF at all time points tested and the increase in area appeared to peak at 4 weeks on HF diet, but did not increase further (Fig 10*A*). Gene expression of both pro- and anti-inflammatory markers, including *IL-1β*, *IL-6*, *TNFα*, *CXCL1*, *F4/80*, *ARG* and *ITgax* in the liver showed no differences at 3 days, 1 week or 16 weeks on a HF diet (data not shown). *IL-10* expression was not detectable in the liver (data not shown). However, *MRC-1*, an anti-inflammatory marker, was down-regulated in HF compared to LF mice after 3 days on diet (Fig 10*B*). *IL-1β* expression showed a difference over time on the LF diet with an up-regulation between 3 days and 16 weeks (Fig 10*C*). *TNFα* did not show an increase in expression over time between 3 days and 16 on the LF diet (Fig 10*D*).

### Inflammatory marker gene expression in muscle

**Figure 11:**
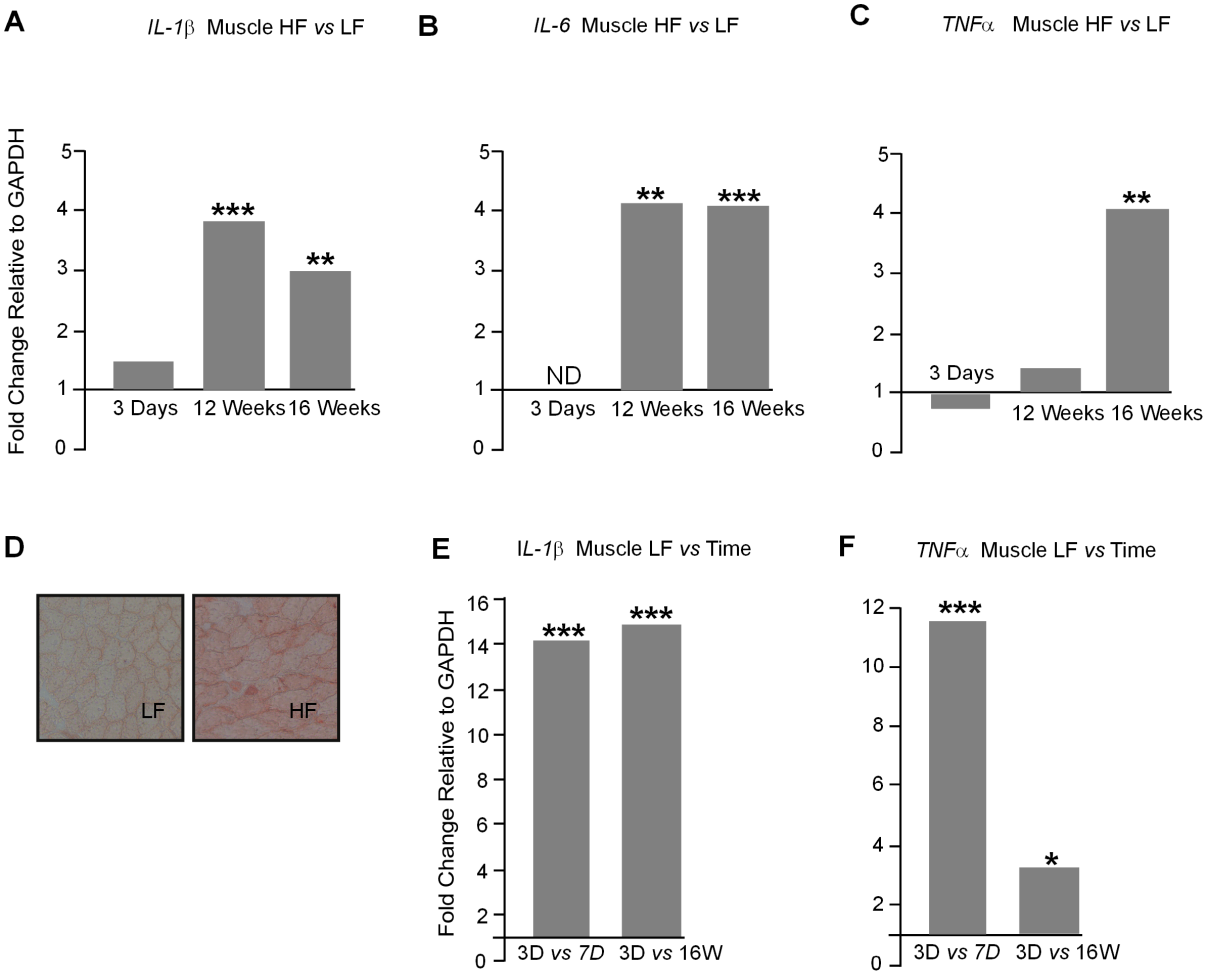
**A.** *IL-1β* gene expression in muscle was unchanged after 3 days of HF diet but was up-regulated at 12 week (*P*<0.001) and 16 weeks (*P*<0.01) **B.** *IL-6* gene expression was not detected (ND) after 3 days on a HF diet but was up-regulated after 12 week (*P*<0.01) and 16 weeks (*P*<0.001) **C.** *TNFα* gene expression was unaffected by a HF diet at 3 days and 12 weeks but was up-regulated at 16 weeks (*P*<0.001) Units are fold expression changes between experimental groups relative to *GAPDH* and calculated from the ΔΔ*C*_t_ values. **D.** Oil red O staining showed an increase in muscle lipid after 12 weeks on HF diet. Mag X 100 (n = 6–8). **E** *IL-1β* gene expression in muscle is strongly up-regulated between 3 days and 1 week (*P*<0.001) and between 3 days and 16 weeks (*P*<0.001) on a LF diet. **F.** *TNFα* gene expression in muscle is up-regulated over time on a LF diet between 3 days and 1 week (*P*<0.001) and between 3 days and 16 weeks (*P*<0.05). Units are fold expression changes between experimental groups relative to *GAPDH* and calculated from the ΔΔ*C*_t_ values (n = 6–8).

Both *IL-1β* and *IL-6* gene expression were up-regulated in muscle at 12 and 16 week in HF compared to LF fed mice (Fig 11 *A*&*B*). *IL-6* gene expression was undetectable after 3 days on diet in both LF and HF fed mice. *TNFα* gene expression showed no difference between HF and LF mice at 3 days and 12 weeks but was increased after 16 weeks (Fig 11*C*). Oil red O staining revealed lipid accumulation in muscle after 12 weeks of HF feeding (Fig 11*D*). Muscle *IL-1β, IL-6* and *TNFα* all showed significant increases in expression on the LF diet with time (Fig 11*E*&*F*). The increase in *IL-6* could not be represented as fold change compared to expression at 3 days as IL-6 gene expression at that time was undetectable (Fig 11*B*).

### Macrophage area and inflammatory marker gene expression in WAT

**Figure 12:**
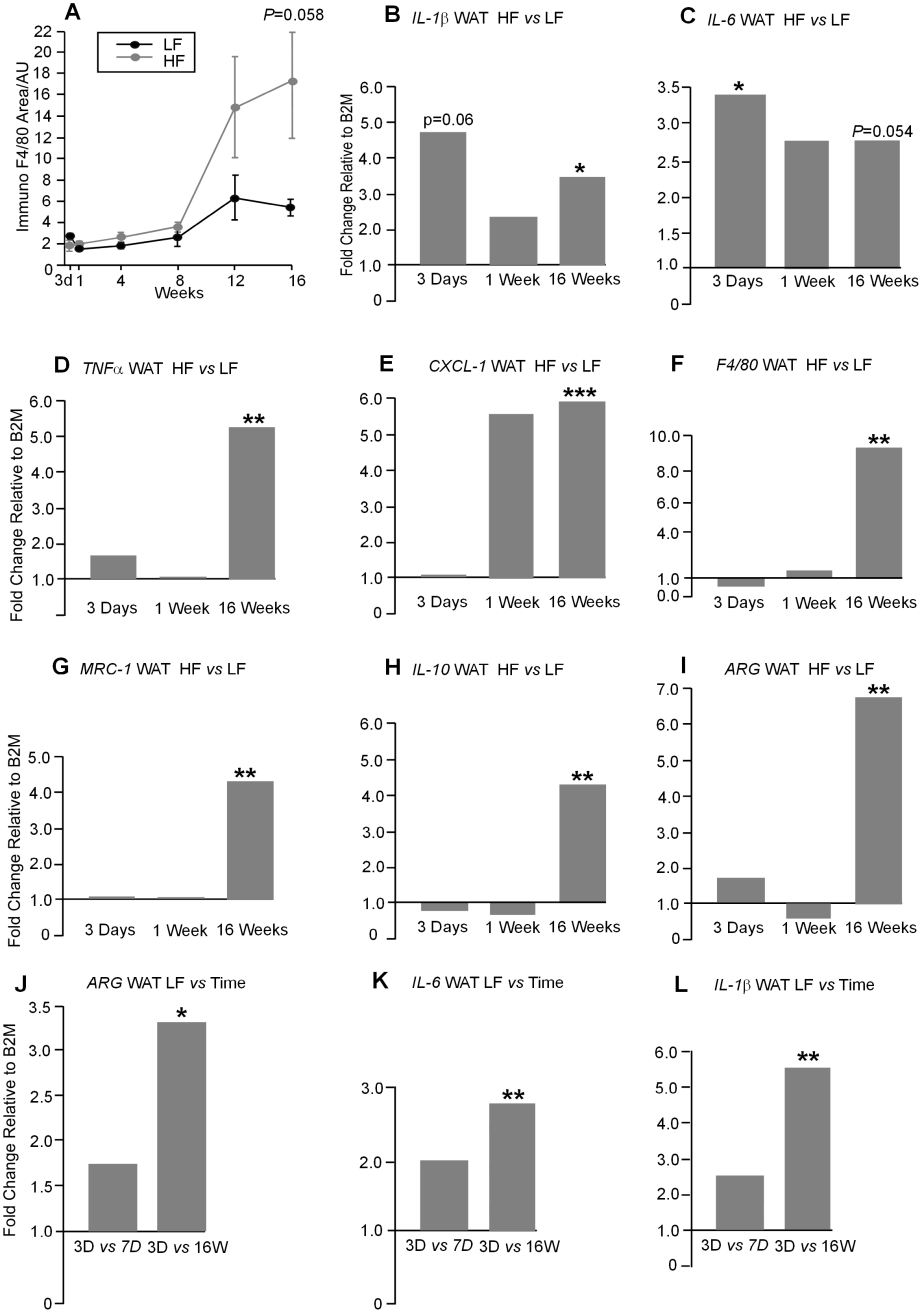
**A.** The area of F4/80 staining in the WAT measured as arbitrary units (AU) was increased in HF fed mice at 12 and 16 weeks compared to the LF diet and almost reached significance at 16 weeks (*P* = 0.058). **B.** *IL-1β* gene expression in white adipose tissue (WAT) was slightly up-regulated after 3 days of HF diet but was also below the level considered to be significant (*P* = 0.06) and was unchanged at 1 week and up-regulated at 16 weeks (*P*<0.05). **C.** *IL-6* gene expression was up-regulated after 3 days on a HF diet (*P*<0.05), unchanged after 1 week and marginally up-regulated at 16 weeks (*P* = 0.054). **D.** *TNFα* gene expression was unchanged after 3 days and 1 week on HF diet but was up-regulated at 16 weeks (*P*<0.001). **E.** *CXCL-1 gene* expression was unchanged at 3 days and 1 week on a HF diet but was up-regulated after 16 weeks (*P*<0.001). **F.** *F4/80* gene expression was unchanged after 3 days and 1 week on a HF diet and up-regulated 16 weeks (*P*<0.01) **G.** *MRC-1* gene expression was unchanged after 3 days and 1 week of HF diet but was up-regulated 16 weeks (*P*<0.01) **H.** *IL-10* gene expression was unchanged after 3 days and 1 week on a HF diet and was up-regulated at 16 weeks (*P*<0.01) **I.** *ARG* gene expression was unchanged after 3 days and 1 week of HF diet but was up-regulated 16 weeks (*P*<0.01). **J** *ARG* gene expression in WAT is unchanged between 3 days and 1 week and up-regulated between 3 days and 16 weeks (*P*<0.05) on a LF diet. **K.** *IL-6* gene expression in WAT is unchanged between 3 days and 1 week and up-regulated between 3 days and 16 weeks on a LF diet (*P*<0.01). **L.** *IL-1β* gene expression in WAT is unchanged between 3 days and 1 week and up-regulated between 3 days and 16 weeks (*P*<0.01) on a LF diet. Units are fold expression changes between experimental groups relative to *B2M* and calculated from the ΔΔ*C*_t_ values (n = 6–8).

Macrophage area identified by F4/80 immunostaining increased after 8 weeks on the HF diet but was highly variable and approached statistical significance after 16 weeks on diet (*P* = 0.058) (Fig 12*A*). A small increase in *IL-1β* was found after 3 days on a HF diet (*P* = 0.06) (Fig 12*B*) and *IL-6* was up-regulated at this time (*P*<0.05) (Fig 12*C*). None of the other genes tested, *TNFα*, *CXCL-1*, *F4/80*, *MRC-1*, *IL-10*, *ARG* showed changes in the HF mice relative to the LF mice after 3 days or 1 week on diet but all genes tested showed an increase in expression after 16 weeks on a HF diet (Fig 12*C–I*). Thus, the majority of these genes showed increases over time on a HF diet (data not shown). *ARG* gene expression showed an increase in LF fed mice over time (*P*<0.05) (12*J*), *IL-6* gene expression was increased on a LF diet (*P*<0.01) (12*K*) and *IL-1β* was significantly increased on a LF diet (*P*<0.01) (12 *L*).

## Discussion

The present results show that the inflammatory response to a HF diet develops in distinct stages; the first occurs within 3 days and correlates with an acute phase response, probably resulting from an acute and transient inflammation and activation of Kupffer cells in the liver, and the second between 12 and 16 weeks on the HF diet occurs when inflammation in WAT and muscle becomes apparent. Between these times i.e. between 1 and 12 weeks on the diet, glucose tolerance remains relatively stable and markers of inflammation are not detectable. Nonetheless, relatively low levels of activation of some markers of inflammation are seen in WAT after 3 days on a HF diet. However, there was no measureable influx of macrophages to WAT at 3 days arguing against a significant role for WAT inflammation at this time. These data are in contrast to studies showing a gradual increase in inflammation and glucose intolerance during the early stages of obesity [19]. The results of the present study point to distinct responses to HF feeding, related initially to nutritional challenge, primarily in the liver and latterly to increased adiposity and increased inflammation in WAT and muscle rather than gradual changes over time. Interestingly, circulating leptin levels are increased at 3 days on a HF diet indicating that the nutritional overload has been recognised [25], but are unchanged after 1 week on a HF diet, although body fat is significantly increased, indicating that nutritional overload, at this time, no longer evokes a leptin response and adiposity hasn't increased sufficiently to do so either.

Data from PF mice at 3 days and 1 week indicate that the relative influence of diet composition and increased caloric intake, on diet-induced changes, differs at these times. Both diet composition and increased caloric intake appear to play roles in increasing body weight, adiposity and liver lipid levels at both 3 days and 1 week after HF feeding. However, diet induced glucose intolerance at 3 days appears to be due to the composition of the HF diet but this changes to caloric intake by 1 week indicating a relatively rapid switch in the mechanism for the induction of insulin insensitivity from dietary fat to overconsumption within this timeframe.

In the present study we utilised plasma proteomics to identify changes in circulating proteins in response to the HF diet and identified numerous changes after 3 days on the HF diet. Surprisingly, most of the proteins identified were indicative of an acute phase response which coincided with the early peak in glucose intolerance. The acute phase response was not apparent after 1 week on HF diet and glucose tolerance also improved at this time. Nonetheless, data from PF mice show that the induction of the acute phase response at 3 days appears to be due to increased caloric intake rather than composition of the HF diet, while glucose intolerance at 3 days was linked to the composition of the diet. These data indicate separate causative mechanisms for these events.

The acute phase response is defined as a systemic change that occurs in response to injury or inflammation, which can be split into two major effects; alterations in the concentration of a number of plasma proteins and certain physiological and behavioural modifications. It plays a fundamental role in avoiding the harmful effects of external challenges and pathogenic events in mammals with the function of re-establishing homeostasis [26] and is principally activated by raised levels of IL-6 [27]. In the present study circulating levels of IL-6 are elevated after 3 days but are not different after 1 week on a HF diet. Nonetheless, circulating levels of IL-1β and TNFα are not different in HF fed mice at any time tested. The values for these two inflammatory markers are highly variable in HF mice, particularly after 3 days on a HF diet, indicating that the timing of their peak values varies between individual animals; a finding which is typical of an acute inflammatory response [26]. The higher levels of circulating IL-6 and changes in a number of acute phase proteins in HF fed mice in the present study are indicative of a rapid and transient inflammatory response to a HF diet that occurs during the first 3 days of feeding. The source of this inflammation is not clearly identifiable as only one anti-inflammatory gene is down-regulated in the liver and two pro-inflammatory genes are marginally up-regulated in WAT. However, the clear increase in Kupffer cell area seen in the liver and the absence of an increase in immunoreactive macrophage area seen in WAT together with previously reported transcriptomic data showing a global increase in pro-inflammatory gene expression in the liver [18] argues for a very early hepatic inflammatory response. This contrasts with both WAT and muscle pro- and anti-inflammatory gene markers which were markedly up-regulated only after 12–16 weeks on HF diet. Nonetheless, gene expression of markers of inflammation in the liver did not increase at any of the times studied despite an increase in total immunoreactive macrophage area in HF fed mice at all the time points tested.

Despite the increase in acute phase proteins in plasma and increased gene expression of acute phase proteins in the liver in the present study, no obvious related behavioural changes were noted in HF fed mice apart from a decrease in food intake to around 85% of the weight of food eaten by LF mice after the first 2 days on diet. This decline in food intake seen in HF fed rodents has been considered as a compensatory mechanism related to an attempt to maintain energy balance, but the results of the present study could point to behavioural changes elicited by the acute phase response. Nonetheless, the intake of the HF fed animals does not increase once the acute phase response subsides indicating that other mechanisms continue to drive this decrease in intake.

In our study the analysis of acute phase proteins in liver and inflammatory marker gene expression in liver, muscle and WAT over time show the importance of age-matched control groups with the expression levels of several genes increasing with time including *IL-1β* in the liver, *ARG, IL-1β* and *IL-6* in WAT and *IL-1β* and *TNFα* in muscle. The changes over time in the muscle are particularly notable with an approximately 10 fold increase in expression over the 16 weeks of the experiment. Lack of appropriate LF controls can result in increases in gene expression with time being inadvertently overlooked or becoming solely attributed to the effects of the HF diet.

In contrast to haptoglobin and alpha-1-antichymotrypsin, the increased level of the putative acute phase protein, ApoA-IV at 1 week is due to both diet composition and increased caloric intake demonstrating a division between ApoA-IV and other acute phase proteins confirmed by the up-regulation of gene expression in the ileum rather than the liver. Higher gene expression of *ApoA-IV* in the ileum in response to a HF diet is not unexpected, as increased chylomicron formation is known to up-regulate the expression of this gene [28]. Several functions have been ascribed to apoA-1V besides lipid absorption including reverse cholesterol transport [29], appetite inhibition [30] and enhanced insulin secretion [31]. Indicating its potential importance in countering HF induced metabolic dysfunction.

The data from the present study, particularly the identification of an early acute phase response, confirms the role of the innate immune system in the initial reaction to HF diet. However, another study using genetically immuno-compromised and macrophage depleted mice also shows early insulin insensitivity [15] indicating that the inflammation and acute phase response may be secondary to the metabolic injury caused by the HF diet and not causative. In comparison, studies using liposome–encapsulated clodronate to deplete Kupffer cells show that the alleviation of early, within 3 days of HF feeding, liver inflammation can lessen the subsequent development of obesity and insulin insensitivity. The results of the present study show that the increase in glucose intolerance after 3 days on the HF diet is due to diet composition, while the acute phase response appears to be due to increased caloric intake, demonstrating that while the two effects are coincidental they are mechanistically unrelated. These data also support the contention that early, within days of HF feeding, insulin insensitivity may occur in the absence of inflammation.

The early peak in glucose intolerance seen in the present study has not been noted in other studies, for example in one study glucose tolerance at 3 days and 10 weeks was described as comparable [15]. There may be differences in experimental approaches that may explain this. Some studies report early hyperphagia seen in the HF fed mice when they were switched from chow to the semi-purified diet [15]. This is almost certainly due to the increased palatability of the semi-purified HF diet which also contains relatively high levels of sucrose. More recently a study reported no discernible increase in insulin insensitivity between 3 days and 12 weeks on HF diet [17] but this study also compared chow with a HF diet and issues of palatability and differences in intake could obscure the relatively high glucose intolerance seen after 3 days on a HF diet in the present study. The comparison of the effects of a semi-purified HF diet with a chow diet rather than with a semi-purified LF diet can show different changes in metabolic parameters [32]. Indeed chow has been found to be protective against the development of adiposity and insulin insensitivity probably due to its high fibre content [33]. Nonetheless, we recognise that in the present study the LF and HF semi-purified diets contain variable amounts of sucrose as well as fat which may lead to metabolic imbalances. The level of sucrose in the LF diet is higher than that in the HF diet so any effects of sucrose alone on plasma glucose levels should be decreased in the HF diet and any metabolic effects seen should be due to the increased fat content of the diet. Even so, this does not discount the fact that dietary fat and sucrose may act synergistically.

The induction of insulin insensitivity seen in humans after intralipid infusion is not accompanied by inflammation [34] contrasting with the increase seen in circulating TNFα after a single HF meal [35]. These studies indicate that both lipid overload and inflammation can play roles in the induction of insulin insensitivity. Nonetheless, in the present study circulating levels of TAG were lower in HF mice and NEFA levels were unchanged. Only LDL and HDL cholesterol levels were higher at 3 days and 16 weeks showing a correlation with the acute phase response, tissue inflammation and glucose intolerance. HDL cholesterol has been shown to be habitually reduced in inflammation and the acute phase response in contrast to the results of the present study [36] demonstrating one factor that makes the inflammation and acute phase response seen after HF feeding in the present study atypical.

In conclusion, a HF diet appears to cause a rapid and transient inflammation which results in an acute phase response, the purpose of which is to limit inflammation induced damage. A HF diet may trigger inflammation directly via activation of TLR4 or indirectly via changes in the gut microbiota and leakage of LPS into the circulation. The data presented in the present study indicate that activation of the innate immune system is a primary event in the development of obesity and metabolic disease [37], as evidenced by an acute phase response, but that this early inflammation is rapidly resolved. The second phase of inflammation in the muscle and WAT causes a more intractable and persistent inflammation which is probably linked to lipid overload and lipotoxicity in WAT and muscle [38] Nonetheless, it remains to be seen how the first phase in inflammation and glucose intolerance contributes to the subsequent development of obesity and insulin insensitivity and whether strains of mice that are less susceptible to a HF diet also mount such a response. This acute reaction to a HF diet in terms of glucose intolerance and the acute phase response may be indicative of a susceptibility to developing obesity and insulin insensitivity.
